# Domain movements of the enhancer-dependent sigma factor drive DNA delivery into the RNA polymerase active site: insights from single molecule studies

**DOI:** 10.1093/nar/gku146

**Published:** 2014-02-19

**Authors:** Amit Sharma, Robert N. Leach, Christopher Gell, Nan Zhang, Patricia C. Burrows, Dale A. Shepherd, Sivaramesh Wigneshweraraj, David Alastair Smith, Xiaodong Zhang, Martin Buck, Peter G. Stockley, Roman Tuma

**Affiliations:** ^1^Astbury Centre for Structural Molecular Biology, University of Leeds, Leeds LS2 9JT, UK, ^2^Department of Life Sciences, Sir Alexander Fleming Building, Imperial College, London SW72AZ, UK and ^3^School of Physics and Astronomy, University of Leeds, Leeds LS2 9JT, UK

## Abstract

Recognition of bacterial promoters is regulated by two distinct classes of sequence-specific sigma factors, σ^70^ or σ^54^, that differ both in their primary sequence and in the requirement of the latter for activation via enhancer-bound upstream activators. The σ^54^ version controls gene expression in response to stress, often mediating pathogenicity. Its activator proteins are members of the AAA+ superfamily and use adenosine triphosphate (ATP) hydrolysis to remodel initially auto-inhibited holoenzyme promoter complexes. We have mapped this remodeling using single-molecule fluorescence spectroscopy. Initial remodeling is nucleotide-independent and driven by binding both ssDNA during promoter melting and activator. However, DNA loading into the RNA polymerase active site depends on co-operative ATP hydrolysis by the activator. Although the coupled promoter recognition and melting steps may be conserved between σ^70^ and σ^54^, the domain movements of the latter have evolved to require an activator ATPase.

## INTRODUCTION

Gene transcription is a tightly regulated cellular process. The basic features of the transcription machinery are conserved through all kingdoms of life, with the multi-subunit RNA polymerase (RNAP) playing a central role (1,2). Bacteria use a sequence-specific DNA-binding protein, the sigma (σ) factor, which, together with five core subunits (α_2_ββ′ω), forms the RNAP holoenzyme that performs promoter recognition and DNA unwinding (3,4) during transcription initiation. Two principal classes of σ factors, the σ^70^ and σ^54^ class, are known. The former mediates transcription of house-keeping genes (σ^70^-RNAP) whereas the latter activates gene expression in response to defined environmental cues (σ^54^-RNAP) (5).

**Figure 1.**
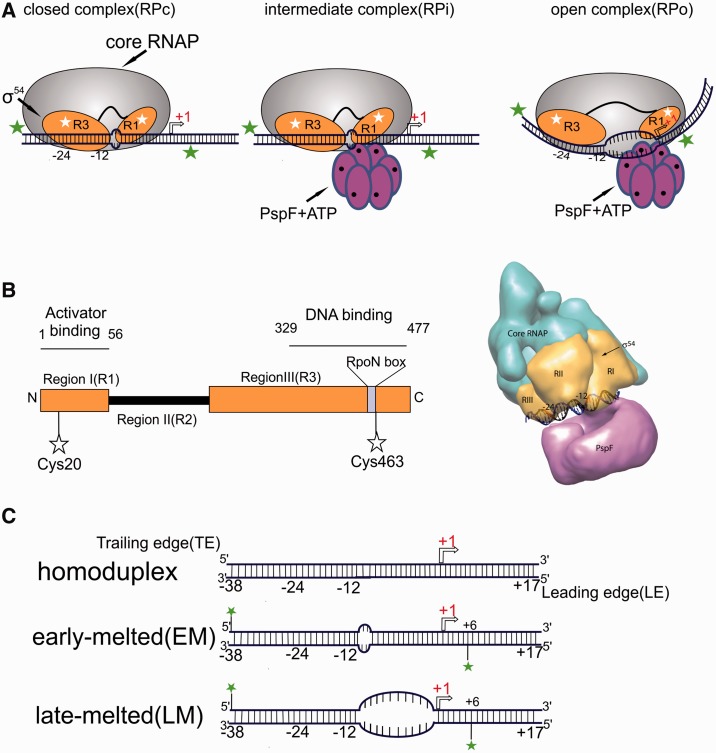
Experimental design and description of components for the smFRET assays. (**A**) Cartoon illustration of the pathway leading to open complex formation in σ^54^-dependent transcription machinery. (**B**) Schematic description of the domain architecture of σ^54^ showing the different domains of the molecule and the positions of dye-labeling (left), and cryo-EM reconstruction of the Eσ^54^:PspF:ADP.AlFx complex depicting the approximate locations of the regions of σ^54^(right). (**C**) Schematic description of *nifH* promoter DNAs used for smFRET assays. Positions of dye-labels used in TIRFM measurements are shown.

On binding to the core RNAP subunits, σ^70^ reorganizes its auto-inhibitory domains, Regions 1.1 and 3.1–4.2, with respect to a central Region 1.2–2.4, leading to the recognition of the −10 promoter element (6) resulting in spontaneous isomerization of the closed promoter complex (RPc) to the open promoter complex (RPo) (3,4). In contrast, the σ^54^-dependent transcription system encounters a kinetic barrier to RPo formation (7). Binding of the RNAP to the promoter at the −12/−24 conserved sequences leads to the formation of a stable RPc that rarely isomerizes spontaneously to the open promoter complex (Figure 1A). Bacterial Enhancer Binding Proteins (bEBPs) belonging to the ATPases associated with various cellular Activities (AAA+) family, such as NtrC and PspF (Phage Shock Protein F), bind to upstream enhancer-binding sequences and couple the energy associated with ATP hydrolysis to remodel the RPc, leading to the formation of the transcriptionally competent RPo (Figure 1A) (8). bEBPs have conserved domain architecture with a central AAA+ domain followed by a HTH DNA binding domain. AAA+ ATPase domains bind to sigma54 via its GAFTGA loop motif (9,10).

Biochemical and mutagenesis experiments have established a number of functional regions of σ^54^ (I, II and III, Figure 1B) and their roles in the various stages of transcription initiation (5). Region III (residues 108–477) is primarily involved in binding to the promoter at several sites, with the strongest interaction being between the RpoN box (residues 454–463) and the consensus −24 GG promoter element (11,12). Region II is variable in amino acid composition across different species and completely absent in *Rhodobacter capsulatus*, suggesting it is nonessential. Region I (residues 1–56) plays a crucial regulatory role in maintaining the RPc by forming a network of interactions both with the −12 promoter element and with Region III, thus preventing spontaneous conversion from RPc to RPo (13–16).

Cryo-EM reconstructions of the σ^54^-holoenzyme revealed the presence of a ‘bridging density’ (Db), believed to be a part of Region I, that occupies the DNA-binding cleft between the β and β′ pincers of the core polymerase, thus potentially preventing loading of the promoter template DNA into the RNAP active site (17). A large-scale domain reorganization of σ^54^ occurs when the holoenzyme interacts with PspF in the presence of ADP.AlFx, suggesting that ATP hydrolysis is key to removal of the ‘roadblock’ posed by the Region I *en route* to RPo formation (17,18). Similarly, the DNaseI footprint of RPc on the *glnAp2* promoter is smaller (−34 to + 2) than that of RPo (− 34 to + 23) (19), suggesting that significant activator-mediated structural reorganization of σ^54^ domains relative to the promoter is also required for productive RPo formation (13,20) through a series of well-orchestrated, but largely unmapped, intermediate steps.

To map these steps, we have used complementary single molecule fluorescence (SMF) techniques, alternating laser excitation (ALEX), total internal reflection fluorescence microscopy (TIRFM) and fluorescence correlation spectroscopy (FCS) to probe the domain movements in σ^54^ on holoenzyme formation, in the closed promoter complex, on binding to a pre-melted *nifH* promoter mimicking the open complex, on PspF-mediated RPi (at the transition state of ATP hydrolysis) and RPo formation and finally on RNA primed initiation and subsequent transcript elongation.

In contrast to σ^70^-RNAP, the σ^54^ enzyme progressively repositions its auto-inhibitory domain in response to binding both single-stranded template DNA and activator. ATP hydrolysis by the latter is then required to fully remove the roadblock to template entering the active site.

## MATERIALS AND METHODS

### Proteins

*Klebsiella pneumoniae* σ^54^ wild-type and variants (C20, C463, C20/C463 and R336A/C20/C463) and the wild-type and T86A versions of the catalytic domain of PspF_(1__–275)_ were expressed and purified as described previously (20). Labeling of the C20/C463 and R336A/C20/C463 dual-Cys variants and the single-Cys variants C20 or C463 of σ^54^ was carried out by addition of a 10-fold molar excess of Alexa Fluor 488 and/or 594-C_5_ maleimide dye over protein, in the presence of 25 mM HEPES 7.4, 100 mM NaCl and 1 mM Tris(2-Carboxy Ethyl) Phosphine (TCEP) at room temperature (∼20–22°C) for ∼2.5 h with further incubation at 4°C overnight. The sample was then quenched with 10 mM β-mercaptoethanol (BME) and kept at 22°C for 15 min. Purification of labeled protein was carried out on a Superdex-75 gel filtration column equilibrated in 25 mM HEPES 7.4, 100 mM NaCl and 10 mM BME. Eluted samples were then analyzed on a 15% (w/v) SDS-PAGE for purity of the labeled species, and the degree of labeling was calculated for each dye using ε = 94 000 cm^−^^1^M^−^^1^for Alexa Fluor 594 and ε = 71 000 cm^−^^1^M^−^^1^ for Alexa Fluor 488 dye. *Escherichia coli* RNA polymerase apo-enzyme was purchased from Epicentre, Inc.

### Promoter DNA template

Synthetic oligonucleotides corresponding to the −38 to + 6 (44 bp) and − 43 to + 17 (60 bp) regions of the *nifH* promoter carrying base pair mismatches at −12/−11 sites to mimic the closed promoter configuration (early-melted), and mismatched between −10 to −1 sites to resemble the open promoter (late-melted) were purchased from IDT DNA(Coralville, USA) (Figure 1C) (21). For TIRF measurements, the − 38 to + 6 oligo was used with Alexa Fluor 488 (Life Technologies Ltd., Paisley, UK) or biotin labels at the 5′-end. ALEX and FCS measurements were carried out on the 60-bp oligonucleotide. Duplex *nifH* promoter DNA was formed by mixing and heating the appropriate templates at 95°C for 5 min, followed by cooling to 22°C.

### Short-primed RNA assay

Functional analysis of labeled σ^54^ was carried out as described in (18).

### ATPase assay

The ATPase activity of PspF_1__–275_ was measured in the presence of Eσ^54^ and late-melted *nifH* promoter (−10 to −1/wt) using the NADH-coupled ATPase assay (22). In all, 100 nM holoenzyme (Eσ^54^) was mixed with 200 nM late-melted *nifH* promoter and 10 μM PspF_1__–275_ and varying concentrations of ATP (0.1–5 mM) at 30°C in the presence of 25 mM Tris (pH 8.0), 100 mM KCl, 10 mM MgCl_2_, 1 mM DTT, 10 U/ml pyruvate kinase, 10 mM phosphoenolpyruvate, 20 U/ml lactate dehydrogenase and 1 mM NADH. Data points were collected for 120 min, and the rate of steady-state ATP hydrolysis calculated by observing the change in A_340 nm_ after normalization with PspF concentration.

### Fluorescence anisotropy

Single-Cys variants of σ^54^(C463 or C20) labeled with either AF488 or 594 dye were used for anisotropy measurements on a Horiba Jobin–Yvon Fluorolog at concentrations identical to that used for ALEX measurements before dilution and at a temperature of 30°C. A_ex_ = 590 nm A_em_ = 617 nm for AF594, D_ex_ = 488 nm D_em_ = 517 nm for AF488). Anisotropy was calculated using the equation r = (I_vv_ − G*I_vh_)/(I_vv_ + 2*G*I_vh_). The polarization correction factor G was determined using horizontal polarization excitation.

### Binding affinity determination

In all experiments, 100 nM of apoenzyme (E) was mixed with 10 nM of dual-labeled σ^54^ (DL) in 1× STA buffer (25 mM Tris acetate, pH 8.0, 8 mM Mg Acetate, 10 mM KCl, 1 mM DTT and 3.5% (w/v) poly-ethylene glycol 6000) (17) and allowed to incubate for 5 min at 30°C. The sample was then excited at 480 nm and an emission spectrum (510–650 nm) collected with the Ex/Em slits set to 5 and 10 nm, respectively. The solution was titrated with unlabeled promoter DNA, and the spectra were recorded at 30°C, allowing an ∼5-min equilibration period after each addition. Fluorescence intensities were corrected for dilution and normalized with respect to the maximum donor fluorescence. Normalized intensities were plotted as a function of promoter concentration and fitted using a single site-model and the non-linear least-squares method in GraphPad Prism (GraphPad Software, www.graphpad.com).

### FCS data collection and processing

FCS measurements were performed as described in (23) on transcription complexes formed on the 60 bp length of Alexa488 labeled early- or late-melted *nifH* promoter at 30°C. Transcription complexes were formed on 1 nM early- or late-melted *nifH* with concentrations of the other components kept identical to that used in the ALEX measurements. AF488-labeled C463 σ^54^ was used to measure the hydrodynamic radius of the holoenzyme.

### Sample preparation and ALEX measurements

For observation of dual-labeled σ^54^ alone, 1 μM protein was diluted into 1× STA buffer (17) supplemented with 0.1 mg/ml BSA to a final concentration of 100 pM. Holoenzyme (Eσ^54^) was prepared by mixing 100 nM dual-labeled σ^54^ with 200 nM core polymerase (Epicentre, Inc.) and incubating for 5 min at 37°C. The effects of holoenzyme binding to early- or late-melted *nifH* promoter DNA (60 bp) were assessed by mixing 100 nM of either oligo to the holoenzyme and incubation of the reaction at 37°C for 5 min. The intermediate trapped complex was prepared by the addition of 0.4 mM ADP.AlFx to the reaction mixture carrying Eσ^54^, PspF_1__–275_ and early- or late-melted *nifH* promoter (24). The effects of ATP hydrolysis on RPc were assessed by the addition of 3 mM ATP to the transcription mix carrying the holoenzyme and early-melted promoter at concentrations mentioned above. RPo formation was investigated at various ATP concentrations in the presence of holoenzyme and late-melted promoter after incubation at 37°C for 30 min; subsequently, the sample was diluted into buffer carrying heparin (0.1 mg/ml) for 1 min. All the above-mentioned reactions were diluted to a final concentration of 100 pM labeled σ^54^ in 1× STA buffer carrying an excess of the other reaction components and 0.1 mg/ml BSA.

Abortive initiation and elongation reactions were carried out by addition of 0.5 mM UpG dinucleotide primer and GTP(0.25 mM) to the activated RPo to form the initial transcribing complex(RP_itc,4_), or the full complement of NTPs (GTP/CTP/UTP/ATP) to form the elongation complex. Samples were incubated at 37°C for 5–7 min to allow formation of these complexes. These were diluted to a final concentration of 100 pM labeled species in 1× STA buffer carrying an excess of the other reaction components and 0.1 mg/ml BSA.

### Alternating laser excitation system and data processing

A custom-made inverted confocal microscope setup coupled with two lasers, a diode-pumped 488 nm laser and a He-Ne 594 nm laser were used to provide excitation at both donor and acceptor fluorophore wavelengths to study FRET. Alternation of laser excitation was achieved by electro-optical modulators that are controlled by software developed in the LabView graphical programming environment (LabView™ 7.1 Professional Development System for Windows, National Instruments, Austin TX, USA) (25). After passing through the polarizers, a spatial filter comprising two 50-mm lenses and a 15-μm pinhole was used to obtain Gaussian beam profiles. A series of mirrors guide the beams to a dual band dichroic mirror that reflects it into the objective. Sample contained in the confocal volume is illuminated by alternating lasers, and the fluorescence signal from the sample is guided through pinhole and filters onto two avalanche photodiodes(APD) where a TTL-type signal is produced for photon counting.

The laser alternation period was set to 100 μs (duty cycle of 40%) with intensity for the 488-nm laser (D_ex_) at ∼90 µW and the 594-nm laser intensity (A_ex_) set to ∼60 μW (continuous wave mode). As each labeled molecule passes through the confocal volume, a single fluorescence burst is generated, which is used to generate four photon streams: 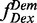, 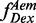, 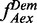, 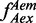, where 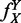 represents the photon count for a single fluorescence burst on excitation at wavelength X (acceptor excitation (Aex) or donor excitation (Dex)) resulting in emission at a wavelength Y (acceptor emission(Aem) or donor emission (Dem)) (26). In our experiments the identification of bursts was performed by setting the threshold for burst selection to ∼3 kHz, and the number of photons constituting a burst to 35–50 photons to remove any false positives due to background. After the identification of bursts, the uncorrected ratiometric observables E and S are calculated using the following equations (25):
(1)
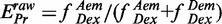

(2)
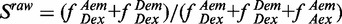



A 2D 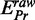-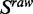 histogram is then plotted and the donor leakage factor (*Lk*) = 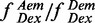 is computed using 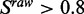, and the direct excitation factor (*Dir*) = 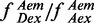 is calculated using 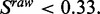 The values for 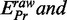 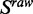 are then subtracted for cross-talk contributions from donor-leakage (*Lk*) and direct excitation of the acceptor (*Dir*) to provide the cross-talk-corrected 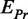 and *S* using the equations:
(3)
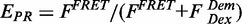

(4)
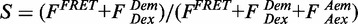

where, 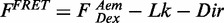


By plotting the 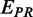 versus 1/*S* for two standard poly-proline chains (6 or 12 residues long) with AF488 and AF594 dyes conjugated to the ends, one obtains the slope(Σ) and the intercept (Ω) from which the detection-correction factor (γ) is derived (eq. 5) (25):
(5)
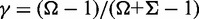



The detection–correction factor γ was found to vary between 0.75–1.2 in this work (27). Finally, the accurate FRET efficiency E is calculated using the following equation (25):
(6)
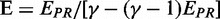



All data collection and processing scripts were coded in LabView graphical programming environment (LabView™ 7.1 Professional Development System for Windows, National Instruments, USA) and Origin 7.0, and were provided to us by Dr. Ted Laurence, Lawrence Berkeley National Laboratories, USA (25).

### Immobilization of transcription complexes and TIRF measurement

Glass coverslip surfaces (BDH Laboratory Supplies, Poole, UK) were prepared following the method described in (28). Coverslips were incubated with Vectabond reagent (Vector Laboratories, Burlingame, USA) according to the manufacturer’s instructions after which they were coated with 25% (w/v) polyethyleneglycol succinimidyl ester (PEG-NHS, Rapp Polymere, Tubingen, Germany) and 0.25% (w/v) biotinylated-PEG-NHS (Rapp Polymere) in 0.1 M sodium bicarbonate (pH 8.3) for ≥3 h to eliminate non-specific adsorption of proteins. The functionalized surface was then rinsed with 10 mM Tris, pH 7.4, and incubated with 0.2 mg/ml Immunopure-streptavidin (Pierce Biotechnology, Rockford, USA) in the same buffer for ≥1 h and then rinsed with 3 × 400 µl of buffer A (25 mM Tris acetate, pH 8.0, with 8 mM magnesium acetate, 10 mM KCl, 1 mM dithiothreitol and 3.5% (w/v) poly-ethylene glycol 6000). Excess buffer was blotted off and Alexa 488-labeled, biotinylated DNA (10 pM in buffer A) was applied to the surface and allowed to bind for 30 min. Excess unbound DNA was then removed by blotting and rinsing using 3 × 400 µl of buffer A.

Transcription complexes were assembled as described in (20) before being diluted to a final concentration of 100 pM in buffer A supplemented with an anti-photobleaching cocktail onto the DNA-derivatised surface. Use of the nucleotide hydrolysis transition state analogue ADPAlF_x_ creates activator bound species described as ‘trapped’ (16), allowing study of a putative intermediate complex (IC) equivalent to those studied by cryoEM (17,29).

Images were acquired using a custom-built inverted microscope with objective illumination (30) and processed using an in-house developed software.

### TIRF data acquisition

Alexa488-DNA fluorescence was excited by 488-nm light in a through-the-objective total internal reflection (TIR) scheme. Our custom-built microscope, described fully in (30), operated in the following manner: laser light (∼3 mW) was focused on the back focal plane of a ×100 1.45 numerical aperture oil immersion microscope objective (Alpha-Plan Fluar, Zeiss). The emerging light was incident on the coverslip/sample interface above the critical angle for TIR, generating an evanescent field that efficiently excites labeled molecules near (<250 nm) the surface. The combined fluorescence emission from the fluorescent dyes, Alexa488 (direct excitation, the donor dye) and Alexa594 (due to FRET, the acceptor dye), was collected by the same objective. The light was then separated by a short-pass dichroic mirror (555DCSX, all filters Chroma Technology Corp., USA). The donor and acceptor emission signals were then filtered (HQ525/50M and HQ620/30M, respectively) and brought back into close, but not overlapping, spatial registration using a long-pass dichroic mirror (565DCLP). The two signals were then focused onto opposite quadrants of the same camera (iXon EMCCD, Andor). The camera was operated at maximum gain, at a temperature of −70°C, with an exposure time of 100 ms and frame-transfer activated. Movies were recorded full-frame from the camera and streamed for up to 60 s.

### TIRF data analysis

Movies were then exported and analyzed using algorithms written in Igor Pro (Wavemetrics, USA): complexes of Alexa488-late-melted promoter with Alexa594-σ^54^-RNAP were identified by manually selecting red spots of fluorescence from the acceptor molecules. The trajectories of fluorescence intensity versus time for these spots (averaged over a 3 × 3 matrix of pixels) and for the corresponding region in the green image (for which a known offset exists) were then examined for single-step photo-bleaching. Up to 50% out of all fluorescent features in the images demonstrated this behavior. The two fluorescence intensity trajectories from each pair of donor and acceptor molecules were then extracted and saved for further analysis.

The FRET efficiency corresponding to a given molecule was obtained from each pair of recorded trajectories in the following manner: first, the background levels (*B_d_* and *B_a_* for the donor and acceptor, respectively) were calculated by averaging over several seconds of the data present at the end of the traces, i.e. after both dyes had photobleached and these values subtracted from each trajectory. Next, the fluorescence signal of donor dye, *I_d_*, for complex without photoactive acceptor dye was obtained by averaging over the donor trajectory after the acceptor has bleached. Finally, the donor signal in the presence of the acceptor, *I_da_*, was determined by averaging over a region in the donor trajectory after the laser was turned on but before the acceptor bleaches. In our experiments ∼50% of trajectories exhibiting the single-step donor photobleaching showed this useful bleaching sequence. Note that because of the long lifetime of the complexes studied here, the disappearance of acceptor signal in trajectories represents acceptor bleaching, rather than complex disassociation, with very high probability. Typically, at least several seconds of the trajectories could be averaged to determine *I_d_* and *I_da_* for each molecule, essentially eliminating shot noise effects. The FRET efficiency was then calculated using the donor-bleaching method (30–32).
(7)
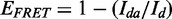



FRET efficiency values were calculated for >100 molecules obtained from at least three separate surface regions for assays with the same components. Histograms (bin size 0.025) were then generated from the FRET efficiency (all analyses carried out in Igor Pro). FRET histograms were then fitted to a function comprising the sum of up to two Gaussian functions of the form:
(8)
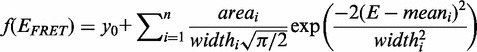

where the number of species fit, *n*, was either 1 or 2. The *mean*, *width* and *area* are the mean FRET efficiency, the width and the area from the Gaussian fit to species *i*, respectively; *y_0_* is the baseline. All errors quoted are the fit errors (± 1 S.D.). Akaike’s Information Criterion Test (Origin 8.6™) was used to determine the statistically relevant number of Gaussians for fitting.

## RESULTS

### Experimental design and description of probes

To investigate domain-specific spatial reorganizations within σ^54^ during the molecular events leading to activator-driven RPo formation and subsequent transcription initiation, we introduced cysteine residues at positions Q20 (in the activator-binding domain, Region I) and E463 (in the DNA-binding domain, Region III) (20) and labeled these sites with donor (Alexa Fluor 488, AF488) and acceptor (Alexa Fluor 594, AF594) fluorophores (Figure 1B, see ‘Materials and Methods’ section and Supplementary Figure S1A). Unlabeled 60-bp DNA fragments of the test *nifH* promoter (Figure 1C and Supplementary Table S1), mimicking the conformations in the closed (RPc, two bases unpaired downstream of the promoter GC) or open (RPo, unpaired for −10 to −1) promoter complexes were used as DNA templates (20). The DL σ^54^ was functionally similar to the wild-type Eσ^54^ holoenzyme (18,33), confirming that the dyes do not interfere significantly with its activity (Supplementary Figure S1B). Anisotropy measurements established that rotational freedom of the dyes was not impeded and that restraining interactions are unlikely (Supplementary Table S2), allowing conversion of FRET efficiencies into relative dye separations.

The binding affinities of the DL Eσ^54^ to the early- and late-melted promoters (60-bp early- or late-melted *nifH*) was estimated by donor fluorophore quenching under conditions similar to that used in single molecule measurements (Supplementary Figure S1C). The K_d_ values obtained for DL Eσ^54^ binding to the early-melted promoter (0.22 ± 0.04 nM) and to the late-melted promoter (0.25 ± 0.03 nM) are similar to that obtained previously for the binding of the unlabeled Eσ^54^ to the homoduplex *nifH* promoter [0.85 nM (34)]. In all the solution single molecule measurements, DL was present at 100 pM, whereas all the other components (including the *nifH* promoter) were in at least 1000-fold molar excess. Thus, according to the measured equilibrium constants, >99% of all DL Eσ^54^ was in the desired complex. Additional confirmation of complex formation was obtained by monitoring the hydrodynamic radius of species in solution using single-molecule fluorescence correlation spectroscopy (smFCS) (23) of singly labeled promoter or sigma54 equivalents (Supplementary Table S3).

In addition to looking at inter-domain reorganization between Regions I and III of σ^54^, we also investigated the spatial movement of these domains relative to the leading or trailing edges of the *nifH* promoter fragments. For these investigations, we used single cysteine variants (Q20C or E463C) labeled with Alexa Fluor 594 C_5_-maleimide dye (20). Early- and late-melted promoters (44-bp long) were end-labeled either at the + 6 (leading edge end) or the −38 (trailing edge end) positions with Alexa Fluor 488 (Figure 1C) (20).

### Activator- and DNA-binding domains of sigma54 are in proximity and conformationally ‘locked’ in the RPc

To investigate relative distance changes occurring between the two fluorophores in DL, we used ALEX (26,35) in a confocal microscope setup that allows accumulation of single molecule FRET (smFRET) data from freely diffusing labeled molecules in the solution (Supplementary Figure S2A and B). Supplementary Figure S2C illustrates the reproducibility of the equilibrium data, while Supplementary Figure S2D points out variations in FRET populations due to batch to batch difference in efficiency of the ATP-driven activation, i.e. as a result of the irreversible step.

**Figure 2.**
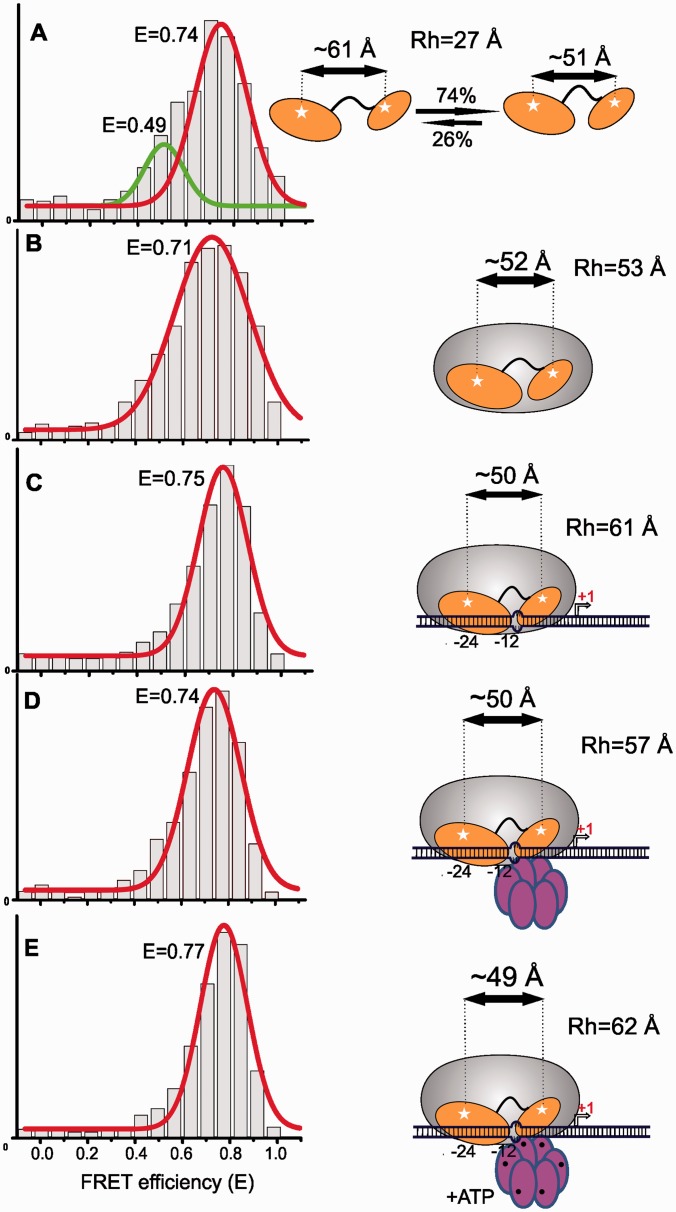
Activator and DNA-binding domains of σ^54^ are in proximity and conformationally ‘locked’ in the RPc. Population histograms probing domain movement of σ^54^ in the closed promoter complex (RPc). (**A**) Dual-labeled σ^54^ alone in solution, (**B**) σ^54^-holoenzyme complex, (**C**) binding of the σ^54^-holoenzyme to the early-melted promoter, (**D**) addition of PspF to the RPc and (**E**) addition of ATP to the RPc.

Small angle X-ray scattering (SAXS) measurements along with cryo-EM of σ^54^ suggest that the molecule is anisometric in its native unbound form (10,36). We first sought to estimate the distance between Regions I and III of DL using smFRET (Figure 2). FRET efficiencies exhibited a bimodal distribution indicating co-existence of two main conformational populations. Fitting to two Gaussian curves is consistent with a major sub-population (73%) with a mean FRET efficiency (E) of 0.74 corresponding to a relative domain separation, R, of ∼51 Å, and a minor sub-population (27%) with E = 0.49 and R of ∼61 Å (Figure 2A and Supplementary Table S3). Native ion-mobility mass spectrometry confirmed the presence of two conformations (Supplementary Figure S3A and B). These FRET signals are from native σ^54^ because in denaturing conditions (6 M urea), E decreased from ∼0.75 to ∼0.08 (Supplementary Figure S3C).

Upon binding to core, the DL FRET showed a single distribution (E = 0.71 and R ∼ 52 Å) close to that of the dominant conformer in free σ^54^ (Figure 2B). Because holoenzyme formation apparently has a relatively small effect, we confirmed that complex formation had occurred using FCS. The hydrodynamic radius (R_h_) of 2.7 nm for Alexa Fluor 488 labeled σ^54^ (Supplementary Table S3) is consistent with the Rg (radius of gyration) value of 3.4 nm derived from SAXS (36). Addition of the core polymerase increased the FCS R_h_ to 5.3 nm, confirming complex formation by >80% of the sigma factor.

During initiation, the holoenzyme (Eσ^54^) binds to the −12/−24 sites on σ^54^-dependent promoters leading to the formation of a transient nucleation site where DNA melting occurs (37). To investigate domain movement during this process, we used the early-melted promoter, a *nifH* duplex having mismatches at −12/−11 mimicking the promoter DNA conformation adopted during formation of the RPc and which is separated from the RPo by a high-energy barrier *in vitro* (15). The RPc showed a single FRET population (E = 0.75 and R ∼ 50.0 Å) similar to the dominant solution conformation, although with a significantly reduced peak width, implying a complex in which the domain positions are more rigidly defined (Figure 2C), consistent with earlier data suggesting that Regions I and III interact with the −24 and −12 sites forming a stable inhibitory complex (14).

Following addition of PspF to the RPc mimic there is little change in the mean FRET efficiency, implying that the inter-domain separation in DL is unchanged (Figure 2D). When 3 mM ATP was added to this mixture there was no change in FRET (Figure 2E), in agreement with previous observations suggesting that the tight binding of σ^54^ at the −12/−11 fork-junction prevents RPo formation *in vitro* (15). We conclude that there is no significant change in the relative positions of Regions I and III of σ^54^ in the RPc mimic, which appear locked into their positions by core. This complex cannot be efficiently remodeled in the context of the RPc. FCS confirmed that complex formation between holoenzyme and template occurred in these samples and was stable to the presence of PspF, with and without ATP (Supplementary Table S3).

### Large-scale remodeling of σ^54^ occurs for RPo formation aided by activator binding

**Figure 3.**
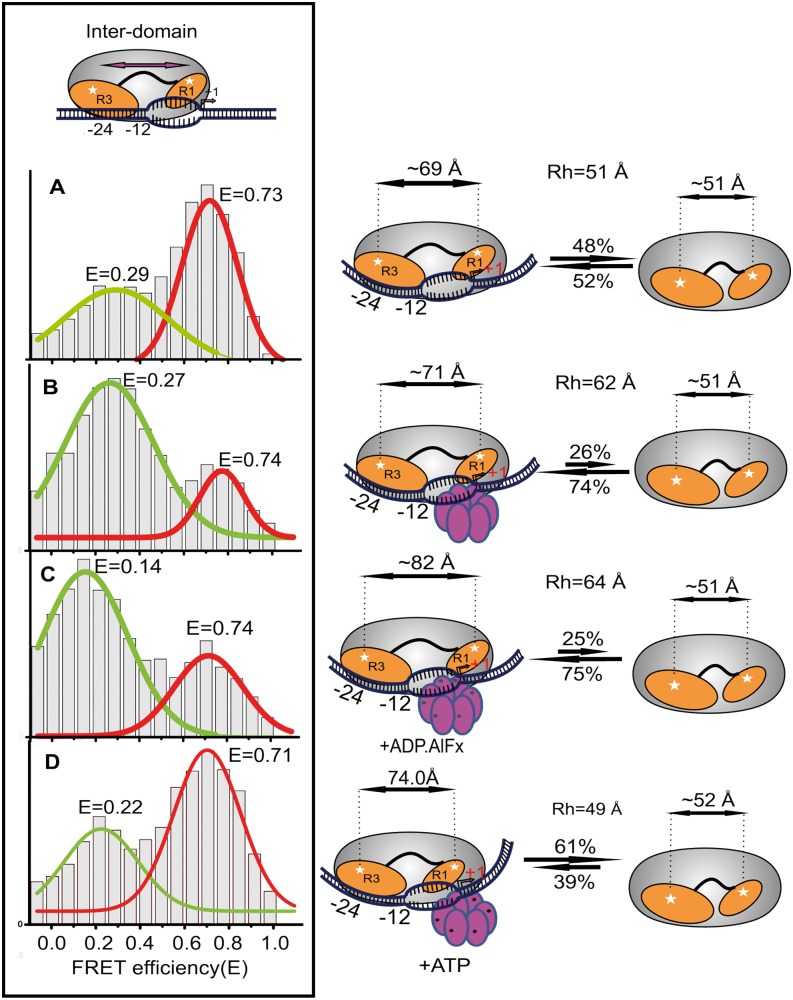
Large-scale remodeling of σ^54^ occurs in events leading to RPo formation*.* Population histograms obtained from ALEX measurements showing change in inter-domain FRET efficiencies in σ^54^. (**A**) On binding to the late-melted *nifH* promoter, (**B**) after addition of PspF, (**C**) After addition of ADP:AlFx and (**D**) After addition of ATP and heparin. Green trace in the panels depicts the newly formed FRET species, whereas the red trace shows the initial conformation adopted by σ^54^.

To gain insights into the σ^54^ reorganization needed for ssDNA delivery to the RNAP active site, we analyzed holoenzyme binding events on the late-melted promoter mimicking the melted DNA within the RPo. Binding of the holoenzyme to this DNA fragment resulted in a bimodal FRET distribution corresponding to two conformations of the DL. These species represent 43% and 57% of the total population, with E = 0.29 and R ∼ 69 Å and E = 0.73 and R ∼ 51 Å, respectively (Figure 3A). The higher FRET species appears similar to the dominant conformer seen for free sigma, but the lower FRET state represents a more extended conformation. The R_h_ value for the Eσ^54^: LM complex is similar to that for Eσ^54^ alone (Supplementary Table S3), but this may reflect the flexibility of a duplex with such an extended single-stranded opening rather than a lack of complex formation given the sub-nanomolar K_d_ values. Clearly, σ^54^ is able to exist in multiple conformations in the holoenzyme, and binding to the late-melted promoter stabilizes a form of σ^54^ in which the relative separation of Regions I and III has increased by ∼18 Å. In the absence of PspF-mediated remodeling, the holoenzyme-late melted promoter should be heparin-sensitive (38) and the low FRET state disappeared when 0.1 mg/ml heparin was added (Supplementary Figure S4A), demonstrating reversibility. These results suggest that partial remodeling of Regions I and III occurs on binding the promoter DNA RPo mimic, presumably because of the recognition of the single-stranded −10 region, but not to the point that the complex is heparin-resistant.

**Figure 4.**
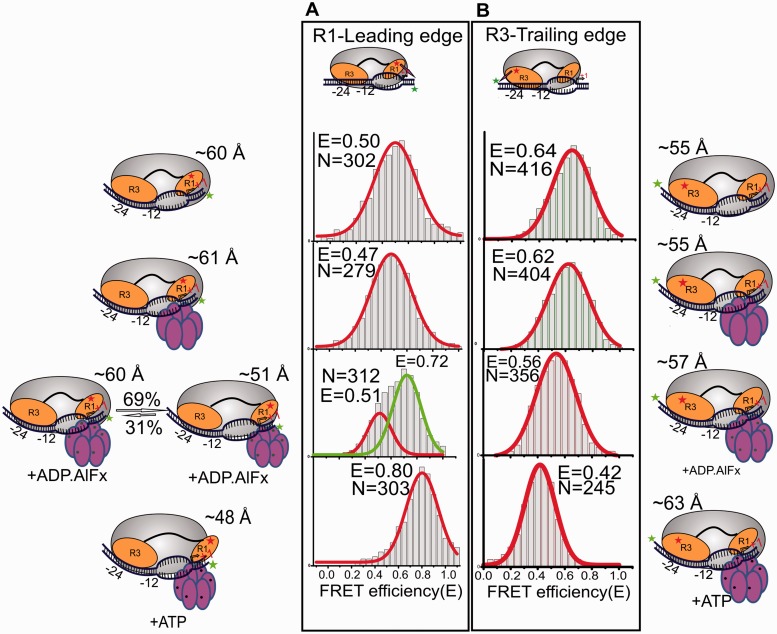
TIRFM FRET measurements showing the relative domain movement of σ^54^ with respect to the leading and the trailing edge of the late-melted promoter DNA. (**A**) The events recorded for the movement of Region I with respect to the leading edge of the late-melted promoter during stages leading to RPo formation. From top to bottom, the histograms show the population distributions obtained on the formation of Eσ^54^:LM complex (top), Eσ^54^:LM:PspF complex (second), Eσ^54^:LM:PspF:ADP.AlFx (third) and Eσ^54^:LM:PspF:ATP with heparin (bottom). (**B**) The population histograms obtained from TIRF measurements for the movement of Region III of σ^54^ with respect to the trailing edge of promoter DNA. Top histogram shows the population distribution obtained on binding of Eσ^54^:LM complex (top), Eσ^54^:LM:PspF complex (second), Eσ^54^:LM:PspF:ADP.AlFx complex (third) and Eσ^54^:LM:PspF:ATP complex with heparin (bottom). The number of events recorded for each experiment is indicated by the value (N).

We then analyzed the effect of adding apo-PspF to the holoenzyme late-melted promoter complex. Although this does not change the FRET efficiencies of the two populations, the lower FRET population (E = 0.27) becomes dominant (78% of the total). This, together with the increased R_h_ of the complex formed (Supplementary Table S3), suggests that the apo-PspF binds and stabilizes the more extended σ^54^ conformation (Figure 3B). This is a novel finding because no stable interactions between apo-PspF and the holoenzyme have been reported previously. To confirm this effect, we used a Loop 1 variant (T86A) of PspF that hydrolyzes ATP but does not interact with Region I, and thus fails to provide mechano-chemical coupling (9). The population histogram of the holoenzyme late-melted promoter in the presence of the T86A PspF variant closely resembles that in the absence of wild-type PspF (Supplementary Figure S4B), implying no productive contact in the absence of T86. Clearly, the PspF Loop1 engages with σ^54^ before any nucleotide-driven transactions. Interestingly, TIRF assays that measure the relative separation of the dyes between each σ^54^ region and the adjacent promoter (trailing or leading) edge do not show bimodal populations or changes in response to these events implying either that they do not alter local DNA contacts before ATP hydrolysis (Figure 4A and B, first and second panels from the top), or that one of the two domains in one conformation is too far from the respective DNA edge to contribute to FRET.

### Nucleotide hydrolysis is required for productive downstream movement of Regions I and III and loads the template strand into the RNAP active site

Because nucleotide hydrolysis by PspF is essential for productive engagement of the template DNA with the DNA-binding cleft of RNAP, we assessed the spatial reorganization of Regions I and III during this step. ADP.AlFx, a nucleotide analog believed to trap the activator at the point of hydrolysis transition state (24), was added to form an intermediate complex, RPi, containing PspF, RNAP, σ^54^ and LM DNA. This led to a further increase in relative domain separation (by ∼10 Å) compared with the nucleotide-free PspF complex (Figure 3C). In contrast to earlier events in which Regions I and III maintained their positions along the promoter DNA, nucleotide analog binding leads to relative movement of Region I toward the leading edge by ∼9 Å (a higher FRET state appearing at E = 0.72, R = 51 Å, Figure 4A third panel). The ratio of high to low FRET states in the TIRF matches that of the two populations seen in solution, consistent with both assays reporting similar molecular events. Region III in this complex remains similarly placed with respect to the trailing edge dye (Figure 4B, third panel). However, these changes do not survive heparin challenge implying that the DNA has not fully moved into the active site and/or RNAP closure around the DNA is incomplete. This is consistent both with the R_h_ value, which barely changes (Supplementary Table S3), and a more limited use of transcription start sites in RPi compared with RPo(18).

In contrast to the RPi, ATP hydrolysis results in a low FRET species at an E = 0.22 that is stable to heparin challenge. The E value suggests that post hydrolysis the domains are slightly closer to each other than in the RPi (Figure 3D). Concomitantly, Region I reorganizes with respect to the leading edge, the change in FRET efficiency suggesting that it moves further downstream (Figure 4A: bottom panel). Region III also relocates with respect to the promoter DNA trailing edge, also moving downstream to be closer to Region I (E = 0.42, Figure 4B, bottom panel). FCS data show that following ATP hydrolysis, the R_h_ of the complex decreases (Supplementary Table S3), consistent with bending of the DNA as the initiation site becomes lodged in the active site of RNAP, similar to observations in the case of the σ^70^ polymerase (39,40).

Activator-mediated nucleotide hydrolysis for RPo formation is efficiently circumnavigated by the R336A variant of σ^54^, which results in transcript formation from the late-melted promoter in the absence of the activator (18,41). The smFRET efficiency with the dual-labeled R336A σ^54^ variant holoenzyme, when bound to the late-melted promoter (Supplementary Figure S6C), was essentially identical (E = 0.23) to that obtained for the wild-type σ^54^ after activator binding and ATP hydrolysis (Figure 3D). This confirms the interpretation that R336A is a bypass mutant for activation-dependent initiation and further establishes that the σ^54^ domain separation is required for this process. The domain disposition in σ^54^R336A alone (Supplementary Figure S6A) or in complex with the core (Supplementary Figure S6B) is similar to that of the wild-type σ^54^ (Figure 2A andB), and only changes following binding to late-melted promoter. Therefore, domain movement during activation must be driven and/or maintained by DNA recognition of the single-stranded promoter DNA element and be coupled to DNA melting. The importance of promoter DNA conformation is further confirmed by the lack of σ^54^ domain movements in the presence of PspF binding and after ATP hydrolysis in either σ^54^ alone or in RNAP holoenzyme in the absence of promoter DNA (Supplementary Figure S7).

We also investigated the dependence of RPo formation on ATP concentration (Supplementary Figure S5) using the appearance of the heparin-resistant domain separation as a measure of remodeling. Although the K_d_ for ATP binding to PspF is ∼0.3 mM (42), similar to the apparent K_M_ ∼ 0.5 mM (Michaelis constant) seen here (Supplementary Figure S5G), the ATP hydrolysis-driven remodeling required higher ATP concentrations (Supplementary Figure S5A–F) yielding an apparent K_M_ of ∼1.5 mM. This suggests that multiple PspF subunits must engage and hydrolyse ATP for productive isomerization to occur, a situation similar to the kinetic cooperativity seen for some hexameric helicases (43).

### The relative positions of Regions I and III remain largely unaltered during early transcription and elongation

**Figure 5.**
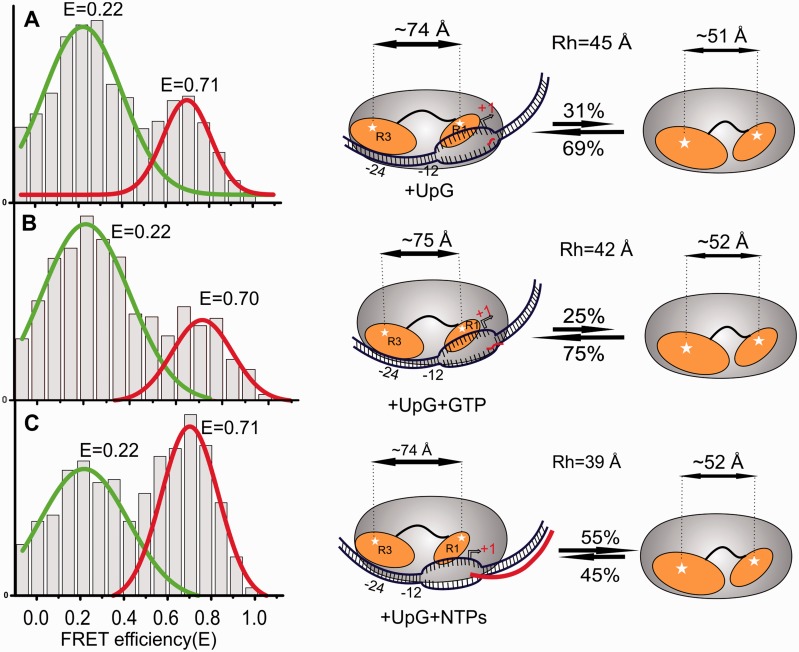
Relative domain positions of σ^54^ remain largely unaltered on abortive transcription and elongation. (**A**) Population histogram showing the change in distance between Regions I and III of σ^54^ on addition of UpG dinucleotide primer to the heparin-resistant RPo, (**B**) On addition of GTP to form the abortive product UpGpGpG and (**C**) on addition of the full complement of rNTPs to the RPo.

Following loading of the template strand and delivery of the +1 start site into the active site of RNA polymerase, the transcriptional machinery is set to form 2–12 nt long abortive transcripts before entering the elongation phase (42,44,45). We used the smFRET assay to investigate the relative domain movement during these processes. We added UpG dinucleotide, the −1+1 primer, to the heparin-resistant RPo. The σ^54^ FRET signals had similar maxima to the two states seen previously in the isomerised RPo (cf. Figure 5A with Figure 3A and Supplementary Figure S5D). Next, a short transcript (UpGpGpG) was formed by addition of GTP (Figure 5B) followed by elongation in the presence of all NTPs (Figure 5C). In both cases the peak FRET efficiencies remain largely unchanged. However, the R_h_ value declines slightly with each addition and transcript extension. This could be due to either partial loss of σ^54^ or to the transcribing template scrunching into the active site. The former explanation is more consistent with the relative increase in the high FRET state, reflecting the dominant conformation of the free sigma (Figure 2A).

## DISCUSSION

**Figure 6.**
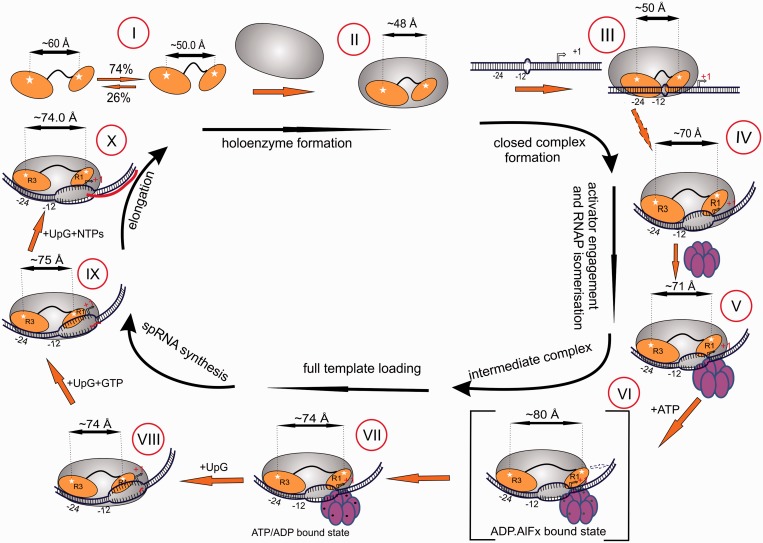
Tracking conformation dynamics of σ^54^ during the transcription cycle. Model shows the domain disposition adopted by the Regions I and III of σ^54^ with respect to each other and also in the context of the *nifH* promoter DNA beginning from free σ^54^ in solution (Step I), holoenzyme formation (Step II), closed promoter complex (RPc, Step III), activator engagement and RNAP isomerization (Steps IV and V), intermediate complex (RPi) formation (Step VI), full template loading (Step VII), initial transcript formation (Steps VIII and IX) and elongation complex (Step X).

Based on previous structural and biochemical work it was anticipated that Regions I and III of σ^54^ undergo significant repositioning within the holoenzyme upon ATP-driven remodeling by PspF (17,20,46). Recently, a multistep kinetic model has been proposed for this activation based on single molecule observations (47). Here, we can complement these studies by assigning the kinetic steps to defined structural rearrangements of Regions I and III with respect to each other, and to the promoter. In accordance with the kinetic scheme (47), the irreversible, ATP hydrolysis-driven step is preceded by two reversible steps. No domain separation occurs during the first step (step II to III, Figure 6), modeled here by Eσ^54^ binding to the early-melted promoter. However, the second step, which is modeled by Eσ^54^-late-melted promoter complex formation, leads to significant separation between Regions I and III (steps III and IV, Figure 6) but without DNA repositioning. Because this domain movement only happens on the partially melted promoter, it is most likely driven by ssDNA recognition, as in the case of σ^70^ where sequence-specific ssDNA binding is coupled to promoter unwinding (3). The domain separation is further stabilized by interaction with the activator PspF even in the absence of ATP (Figure 6, IV and V). In the presence of the transition state analog ADP:AlF_x_ σ^54^ domains become further separated and Region I moves toward the leading edge of the promoter (Figure 6, V and VI). However, only ATP hydrolysis makes the protein rearrangement irreversible (Figure 6, VI to VII), the domain separation decreasing to the one seen in the nucleotide-free state (V in Figure 6) but with both regions closer to the leading edge. This most likely reflects loading of the template DNA strand into the active site, resulting in bending of the promoter and polymerase ββ′ clamp closure (48). DNA footprints of the Eσ^54^ RPc and RPo show little difference at the upstream trailing edge, whereas in RPo the downstream footprint is extended, the interaction with the downstream fork junction is changed and the start site DNA is well within RNAP. Thus, changes in trailing edge FRET with the Region III label may reflect increased upstream wrapping of DNA in RPo, and changes in σ^54^ Regions I to III separations between RPo and RPc are dominated by a movement of Region I relative to a more static Region III.

It is interesting to note that Regions I and III are significantly more separated in the transition state complex (ADP:AlF_x_) than in the final state after ATP hydrolysis. The NtrC1 activator has been shown to convert from a heptamer to a hexamer, which subsequently undergoes a large conformational change in response to cooperative ATP analog binding, yielding an asymmetric gapped hexamer (49). In particular, its GAFTGA loop region responds to binding of different nucleotides. The largest conformational change, as with PspF, occurs with ADP:AlFx (50). Our data show that the pre-hydrolysis transition state of ATP results in a ‘power-stroke’ that moves the Region I further toward the downstream promoter element, most likely due to movement of the GAFTGA loop out of the plane of the hexameric ATPase. In the next step, after release of ADP and/or Pi, Region I moves closer to Region III, presumably due to withdrawal of the GAFTGA loop, and the complex is rendered heparin-insensitive. The hydrodynamic radius of the complex also decreases, suggesting that at this point the template strand is fully loaded into the DNA binding cleft and as a consequence is bent. At this step, polymerase clamp closure also occurs, loading the template strand into the active site and enabling transcription (48,50).

The high K_M_ for the formation of the heparin-resistant complex as judged from the relative separation of Regions I and III is indicative of cooperativity in which several subunits within the PspF hexamer need to bind and/or hydrolyse ATP before performing the mechanical activity. This is consistent with the proposed cooperative formation of gapped hexamer of the related AAA+ activator protein NtrC1 (49). Because ATP hydrolysis repositions both Regions I and III with respect to the promoter DNA and PspF has extensive contact with DNA, it is plausible that multiple ATP binding/hydrolysis events are needed to sequentially remodel σ^54^ domains and reposition DNA.

Once the domains of σ^54^ have reorganized to allow the template strand to access the active site of RNAP, the disposition of Regions I and III remains largely unchanged both in the RPo and within the subsequent transcribing complexes that retain σ^54^.

Finally, we suggest that whereas for σ^70^ proteins where the Region 1.1 is repositioned from the RNAP DNA-binding channel to allow RPo formation (or the RNAP clamp must open further), σ^54^ Region I is actively relocated by the ATPase during hydrolysis. In contrast, for σ^70^ RPo formation, signals from DNA may induce opening and closing of the RNAP clamp, causing Region 1.1 to move away from the RNAP main channel. Hence, for σ^54^ RPo formation is coupled to its cognate enzymatic ATPase, but for σ^70^ to promoter DNA motions.

## SUPPLEMENTARY DATA

Supplementary Data are available at NAR Online.

## FUNDING

BBSRC [BB/H011234/1 to PGS and RT] and [BB/H012249/1 to X.Z., M.B., S.W.]; University of Leeds for supporting the Single Molecule Facility and Wellcome Trust Joint Infrastructure Facility [062164] and [090932/Z/09/Z]; P.C.B. was supported by a Wellcome Trust project grant [WT093044MA to M.B.] and N.Z. was supported by BBSRC project grants [BB/J002828/1 and BB/G001278/1 to M.B.]. Funding for open access charge: BBSRC and RC UK.

*Conflict of interest statement*. None declared.

## Supplementary Material

Supplementary Data
