# Supplementary material for: Domain movements of the enhancer-dependent sigma factor drive DNA delivery into the RNA polymerase active site: insights from single molecule studies
Source: Nucleic Acids Res. 2014 Feb 19;42(8):5177–90. doi: 10.1093/nar/gku146 (PMC4005640; doi:10.1093/nar/gku146)
Supplement: Supplementary Data [file supp_42_8_5177__index.html]

Domain movements of the enhancer-dependent sigma factor drive DNA delivery into the RNA polymerase active site: insights from single molecule studies — Domain movements of the enhancer-dependent sigma factor drive DNA delivery into the RNA polymerase active site: insights from single molecule studies — Supplementary Data 

# Domain movements of the enhancer-dependent sigma factor drive DNA delivery into the RNA polymerase active site: insights from single molecule studies

## Supplementary Data

files

**Files in this Data Supplement:**

- Supplementary Data - pdf file
