# Supplementary material for: Domain movements of the enhancer-dependent sigma factor drive DNA delivery into the RNA polymerase active site: insights from single molecule studies
Source: Nucleic Acids Res. 2014 Feb 19;42(8):5177–90. doi: 10.1093/nar/gku146 (PMC4005640; doi:10.1093/nar/gku146)
Supplement: Supplementary Data [file supp_gku146_nar-03471-h-2013-File008.pdf]

Supplemental Information related to:

Domain movements of the enhancer-dependent sigma factor drive DNA delivery into the RNA polymerase active site: Insights from Single Molecule Studies.

Amit Sharma[1], Robert N. Leach[1a], Christopher Gell[1], Nan Zhang[3], Patricia Burrows[3], Dale A. Shepherd[1b], Sivaramesh Wigneshweraraj[3c], D. Alastair Smith[1,2d], Xiaodong Zhang[3], Martin Buck[3], Peter G. Stockley[1*], and Roman Tuma[1*].

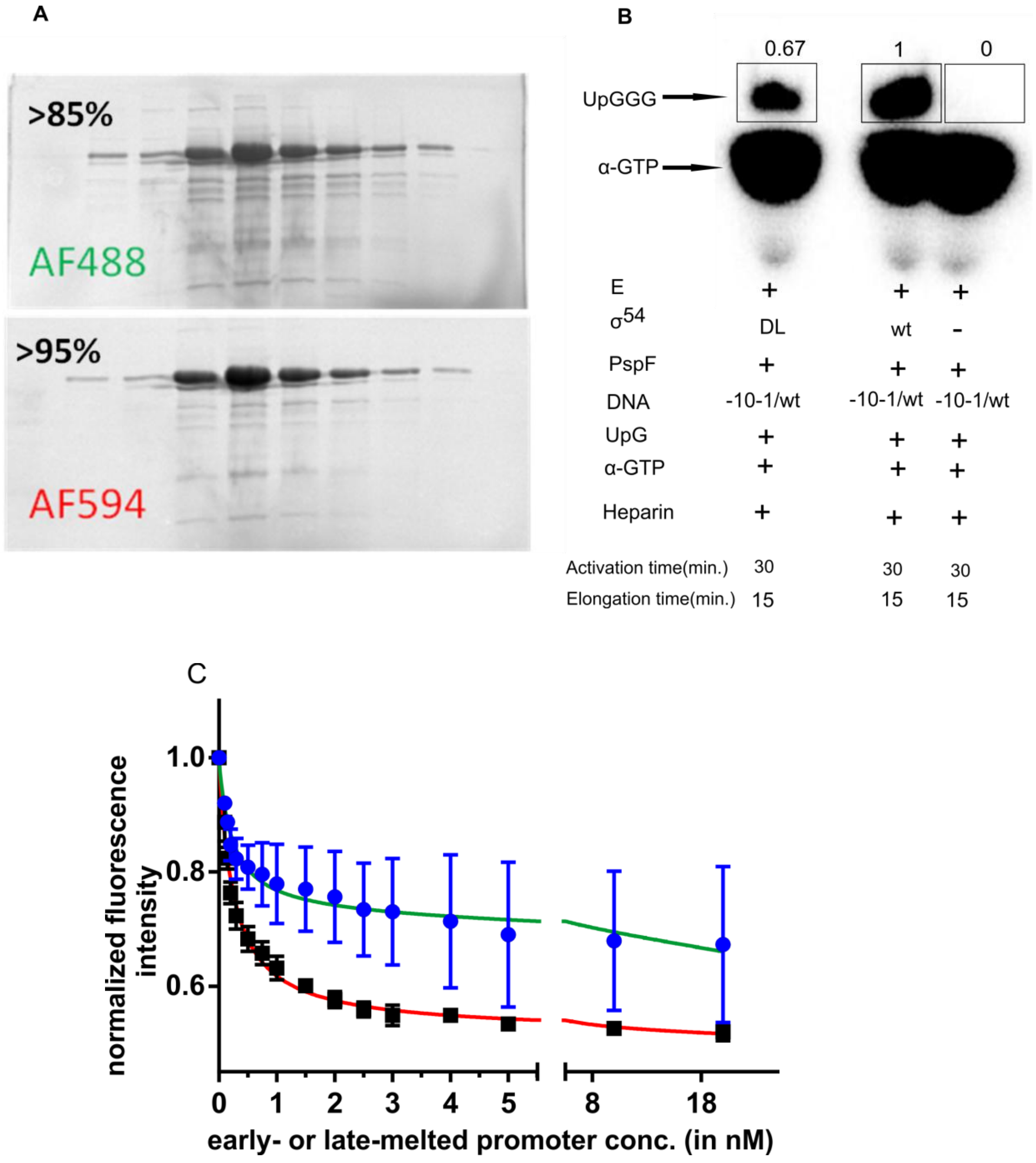

Figure S1: **Labelling and functional analysis of DLσ54**. (A) The double mutant protein labelled with AlexaFluor 488 (AF488) and AlexaFluor 594(AF594) C_5-maleimide dyes (designated DL here) was purified from free dyes by size exclusion chromatography. SDS-PAGE showing the purity and degree of labelling of dual-labeled σ^{54}. The degree of labelling for each dye was above ~85% as judged by UV-VIS absorption spectra. (B) spRNA assay probing the activity of the DL. Urea denaturing gel shows the amount of radio-labeled

spRNA(UpGpGpG) resulting from UpG primed transcription from late-melted *nifH*(-10 to -1/wt) promoter with the DL(first lane). Comparison is made with the amount of spRNA produced by wild-type σ^{54} on the late-melted promoter (second lane). All intensities were quantified using Image J software after background correction and are expressed as a fraction of the amount produced from the wild-type σ^{54} above the boxed bands. (C) Determination of the dissociation constant (K_d) of $E\sigma^{54}$(DL) for the promoter mimics by fluorescence quenching. Normalized donor fluorescence intensities from $E\sigma^{54}$(DL) (10 nM in 1x STA buffer) in the presence of increasing concentrations of early-melted (blue circles) and late-melted (black squares) *nifH* promoter are shown with standard errors estimated from repeated measurements. Donor emission at 517 nm was monitored and the intensities were corrected for dilution and normalized with respect to the initial value. The K_d values estimated by least squares fitting to a single-site binding model (GraphPad) were 0.22±0.04 nM and 0.25±0.03 nM for the early-melted and the late-melted *nifH* promoter, respectively.

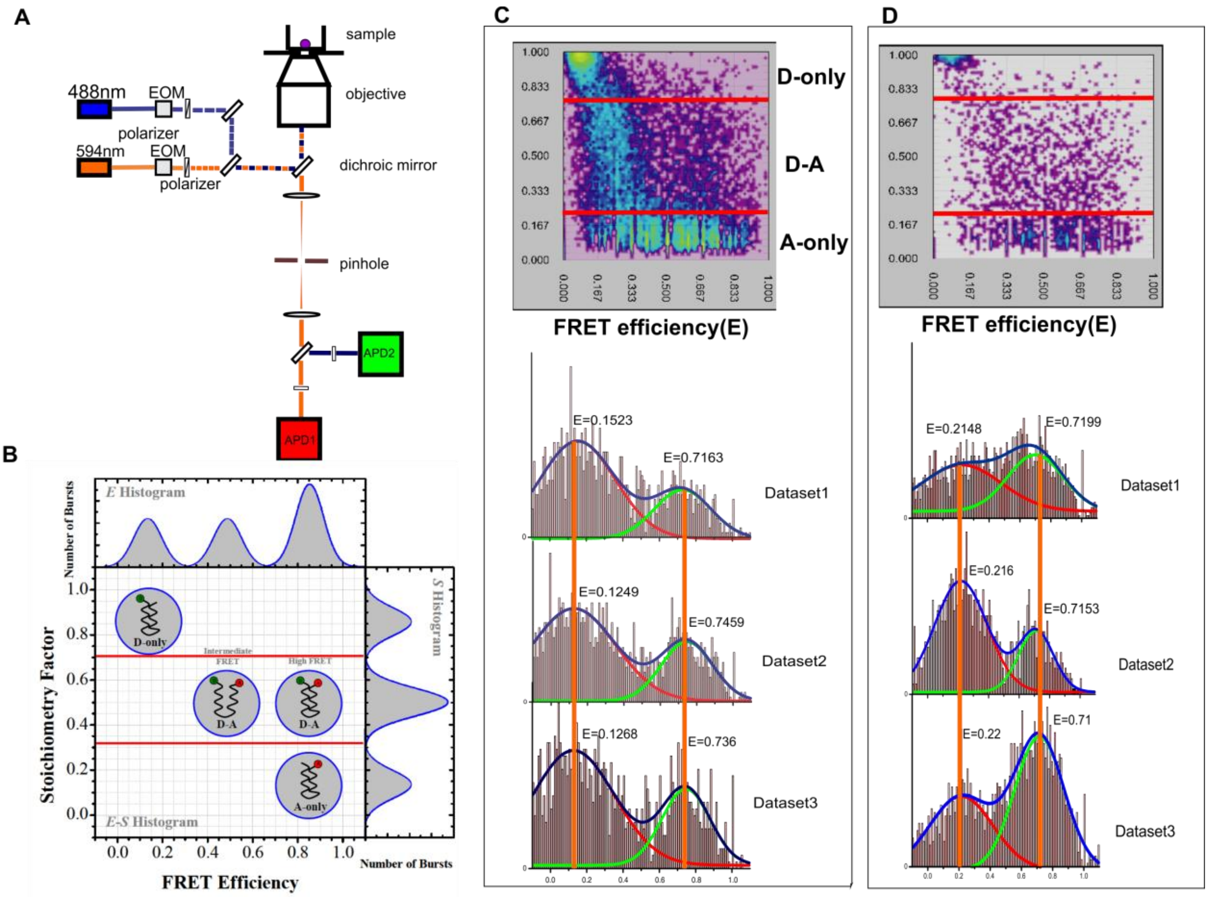

Figure S2: **Principle of Alternating Laser Excitation data acquisition and processing.** (A) Schematic showing the ALEX set-up used for data collection (see Methods). In a confocal set-up, sample at low concentration (typically pM) is illuminated alternately by two electro-optical modulated lasers at 488 nm (donor excitation) and 594 nm (acceptor excitation) and the fluorescent signal is detected by two Avalanche Photo-diodes (APD) after passing through a dichroic mirror, pinhole and series of bandpass filters. (B) Idealised two-dimensional histogram of fluorescent photon bursts classified according to both the ratiometric observable E (FRET efficiency) and S (stoichiometric factor) and showing the regions expected for species carrying only donor (D-only), or acceptor (A-only), or a donor-acceptor pair (D-A). The two-dimensional histogram can be projected onto the x-axis to obtain the FRET efficiency distribution from differently labelled species passing through the confocal volume. Similar projection onto the y-axis gives distribution of the stoichiometry

factor. In this work the two dimensional histograms were zoned (horizontal red lines) to extract FRET efficiency distributions from species labelled with a donor-acceptor pair while ignoring the singly labelled species. (C) E-S distribution of photon bursts obtained after addition of PspF:ADP.AlFx to the $E\sigma^{54}$:LM (top), and the projected FRET efficiency histograms from three independent experiments (bottom). (D) Top panel: E-S distribution of photon bursts observed for the $E\sigma^{54}$:LM: PspF:ATP complex after challenge by heparin. Bottom Panel: projected FRET efficiency histograms for three independent experiments. While FRET efficiencies are reproducible for both conditions the populations in panel (d) vary due to batch to batch variation of the ATP hydrolysis driven irreversible step. This may reflect the stochastic nature of the activation (see Fig 4) during which some events are abortive and the complexes revert back to $E\sigma^{54}$ upon heparin challenge (high FRET peak).

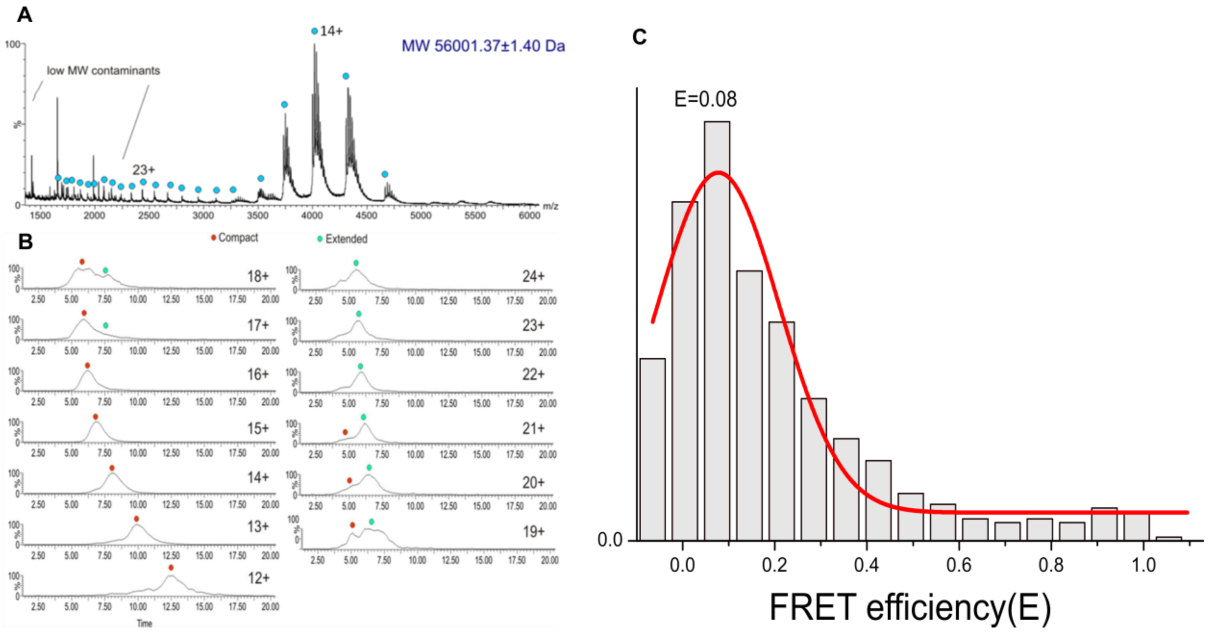

Figure S3: σ^{54} is conformationally heterogeneous with the Region I and III in close proximity. (A) Nano-electrospray ionisation mass spectrum obtained for σ^{54}. The dominant signals in the spectrum correspond to a narrow charge state distribution centred on 14+ charges, characteristic of a compact structure. There are also a number of high charge states up to 33+, characteristic of a more extended structure (NB. the spectrum was acquired under low activation conditions, therefore the low charge state signals are observed with multiple adducts). (B) Arrival time plots of charge states 12+ to 24+ of σ^{54}. The compact conformation (red circles) is the only species at charge states 12+ to 16+. As the charge increases further, an extended conformation (green circles) with a greater arrival time is populated. At 19+ charges the extended conformation dominates and does so upon the addition of subsequent charges. These data illustrate the conformational heterogeneity of σ^{54}. (C) Denaturation of DL σ^{54} in 6M urea shows that the FRET population shifts to a lower FRET efficiency (E =0.08, R ~90 nm), cf Fig. 2A. This suggests that in the natively folded state the Region I and III of σ^{54} are in proximity.

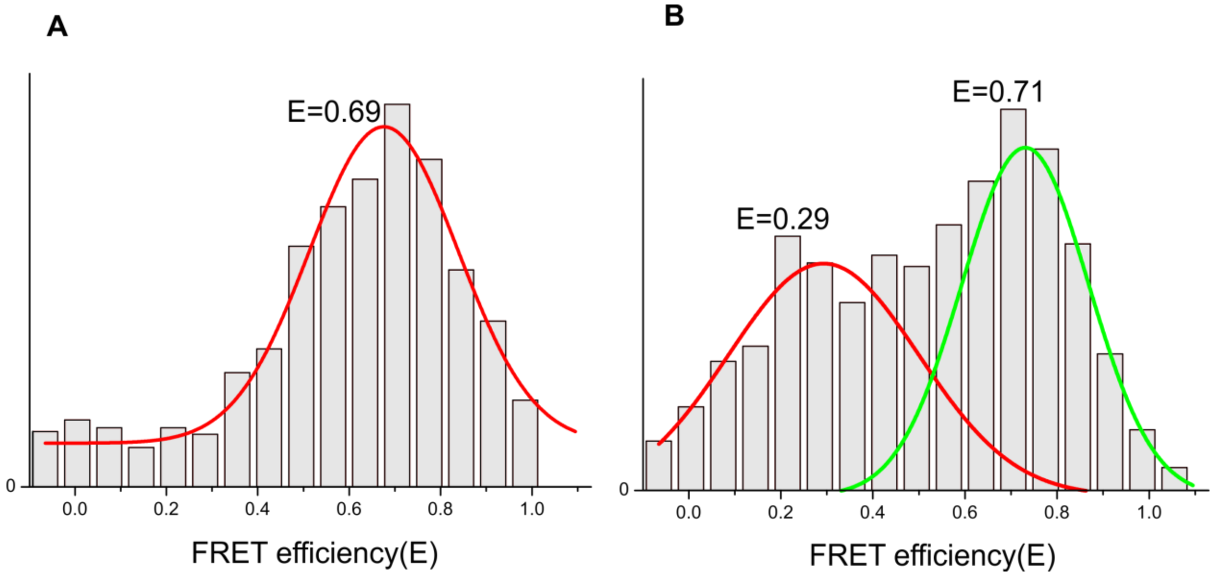

Figure S4: **Eσ^{54}-late-melted promoter complex is heparin sensitive and interaction with PspF facilitates its formation.** (A) Data shows that addition of heparin to the Eσ^{54}-late-melted promoter complex leads to loss of the low FRET population (E=0.29), suggesting that the low FRET conformer (cf. Fig. 3A) is sensitive to heparin prior to PspF addition and ATP hydrolysis. (B) Data shows that addition of the T86A variant of PspF to the holoenzyme-late-melted promoter complex essentially results in a FRET population distribution similar to that of Eσ^{54}-late-melted promoter complex(cf. Fig. 3A), demonstrating that increase in the lower FRET conformer is most likely due to interaction of the GAFTGA loop of PspF with σ^{54}.

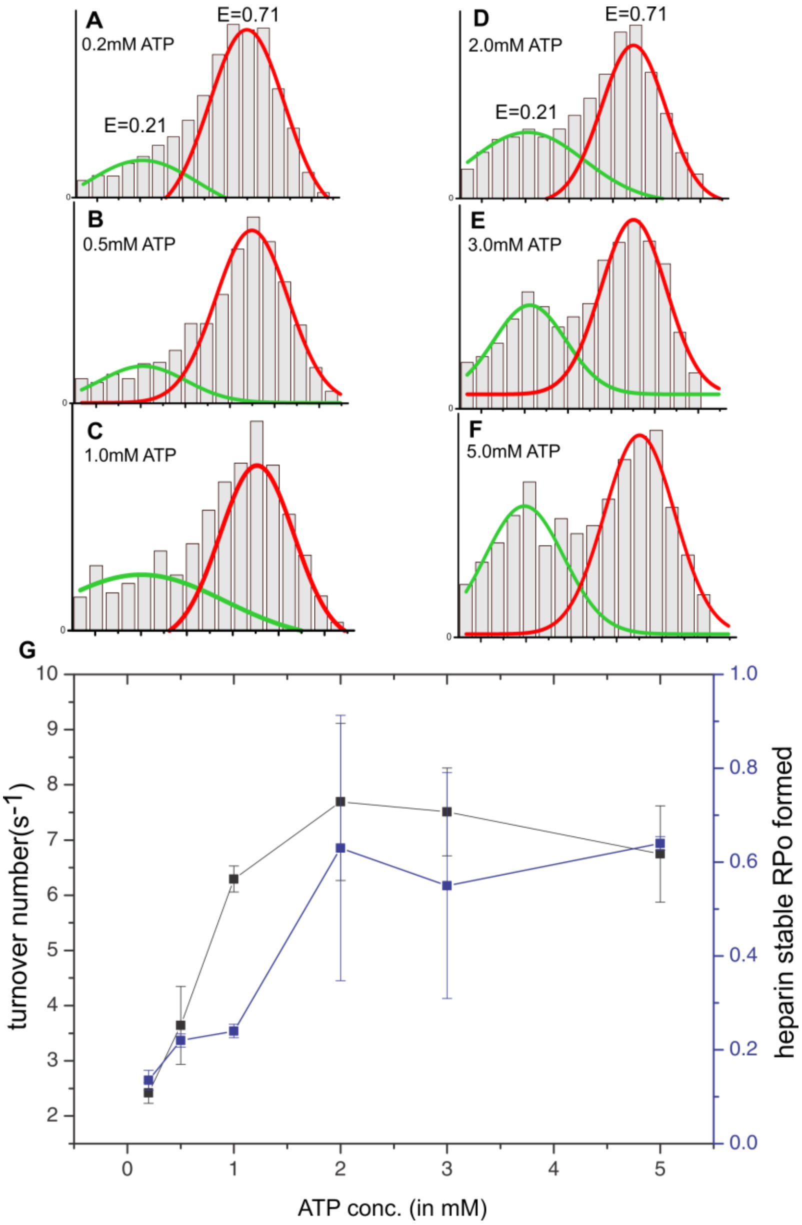

Figure S5: **Multiple PspF subunits must bind ATP for productive RPo formation**. Panel A-F: Population histograms showing the effect of increasing concentrations of ATP on formation of heparin resistant RPo. Two Gaussian curves were fitted to the population with the means set at E=0.22 and 0.71 and the area under the curve was used to deduce the fraction of lower FRET population. (G) Plot showing the comparing the fraction of lower FRET population(representing the heparin-resistant RPo, right-hand y-axis) versus the turnover number of the ATPase- PspF (left-hand y-axis) as a function of ATP concentration. Error bars indicate SD obtained from three independent experiments.

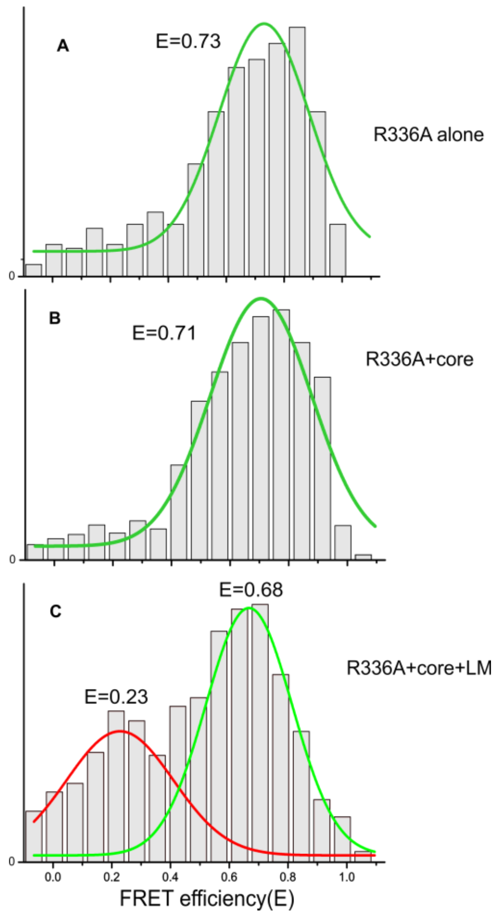

Figure S6: Organization of the bypass-mutant (R336A) Eσ^{54} on the late-melted promoter after heparin challenge is similar to that observed for the RPo formed after activation by PspF. (A) Population histogram obtained from dual-labelled variant (R336A) of σ^{54}. (B) Population histogram obtained from dual-labelled variant(R336A) of σ^{54} in complex with the core polymerase. (C) Population histogram showing the presence of a lower FRET conformer upon binding of the dual-labelled variant(R336A) of Eσ^{54} to the late-melted promoter DNA. The FRET efficiency observed is similar to that achieved for the RPo(cf. Fig. 3D) and different from that obtained after heparin challenge of the Eσ^{54} :LM complex(cf. Supplementary Fig. 4A). This suggests that domains of the bypass mutant of σ^{54} adopt a conformational arrangement that is similar to that observed for the wild-type holoenzyme on the -10-1/wt promoter complex after PspF-dependent ATP hydrolysis and heparin challenge.

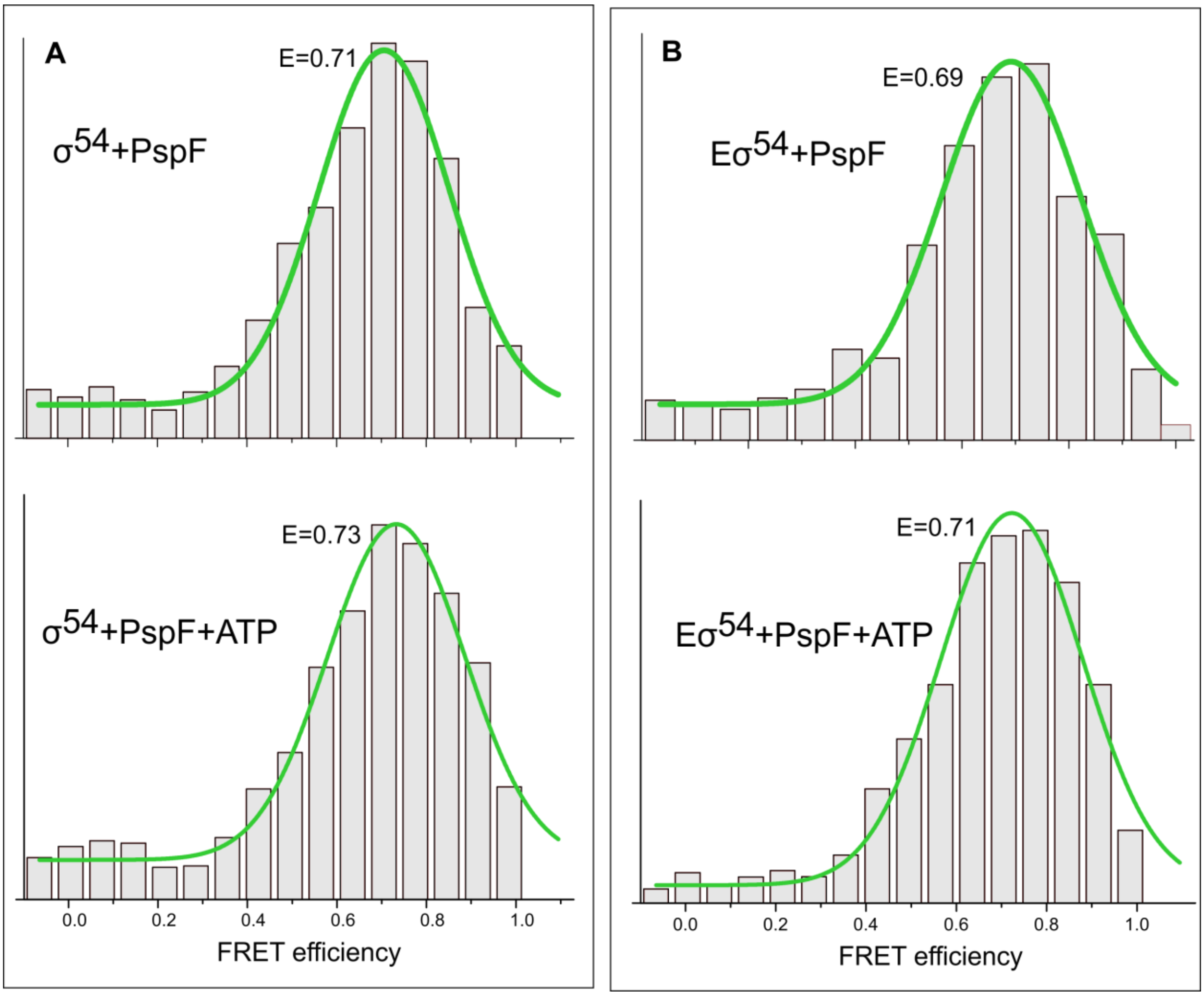

Figure S7: Promoter engagement is obligatory for remodelling of σ^{54}. (A) Top panel shows the FRET population distribution obtained from DL in the presence of excess PspF(10uM). Bottom panel shows the effect of addition of 3mM ATP to the σ^{54}-PspF complex. (B) Top panel shows the FRET population distribution obtained after the addition of excess PspF to the $E\sigma^{54}$ holoenzyme complex. Bottom panel shows the FRET distribution obtained after the activation of the $E\sigma^{54}$-PspF complex by ATP. In either case the addition of activation of PspF by ATP does not result in any significant change in the FRET efficiency, suggesting that promoter binding by the σ^{54} is obligatory for remodelling.

Table S1: **List of oligos used in the study.** List depicts the oligo sequences used in the study, with the regions of mismatch shown in bold italics and the transcription start site marked by a box.

Unmelted *nifH* promoter

-1

5'- TTT TAT TTC AGA CGG CTG GCA CGA CTT TTG CAC GAT CAG CCC TGG GCG CGC ATG CTG TTG-3'
3'-AAA ATA AAG TCT GCC GAC CGT GCT GAA AAC GTG CTA GTC GGG ACC CGC GCG TAC GAC AAC-5'

Early-melted *nifH* promoter

-1

5'- TTT TAT TTC AGA CGG CTG GCA CGA CTT TTG C***CA*** GAT CAG CCC TGG GCG CGC ATG CTG TTG-3'
3'-AAA ATA AAG TCT GCC GAC CGT GCT GAA AAC GTG CTA GTC GGG ACC CGC GCG TAC GAC AAC-5'

Late-melted *nifH* promoter

-1

5'-TTT TAT TTC AGA CGG CTG GCA CGA CTT TTG CAC ***TCG ACT AAA G***GG GCG CGC ATG CTG TTG-3'
3'-AAA ATA AAG TCT GCC GAC CGT GCT GAA AAC GTG CTA GTC GGG ACC CGC GCG TAC GAC AAC-5'

Table S2: **Fluorescence anisotropy measurements of singly-labelled 20C or E463C variant of σ^{54} labelled with AlexaFluor 488 or 594 dyes.** The dual-labelling strategy leads to random attachment of dyes at either Cys residue, which could result in differing molecular environments. Anisotropy measurements of single cysteine variants labelled with either dye show that both dyes have similar tumbling rates at either site during the various stages of RPc and RPo formation, establishing that rotational freedom of the dyes is not impeded and that restrining interactions are unlikely.

Molecular Species	20C-AF594 label	20C-AF488 label	463C-AF594 label	463C-AF488 label
**σ^{54}**	0.26	0.24	0.14	0.22
**Eσ^{54}**	0.25	0.23	0.15	0.24
**Early-melted promoter**				
**Eσ^{54}+EM**	0.28	0.18	0.14	0.22
**Eσ^{54}+EM+PspF**	0.28	0.17	0.16	0.22
**Eσ^{54}+EM+PspF+ATP**	0.29	0.18	0.14	0.21
**Late-melted promoter**				
**Eσ^{54}+LM**	0.28	0.2	0.15	0.23
**Eσ^{54}+LM+PspF**	0.28	0.21	0.16	0.22
**Eσ^{54}+LM+PspF+ATP**	0.25	0.19	0.12	0.18
**RPo+UpG**	0.21	0.2	0.11	0.18
**RPo+UpG+GTP**	0.24	0.20	0.11	0.17
**RPo+UpG+NTP**	0.24	0.20	0.11	0.17

Table S3: **FCS measurements of transcription complexes formed on Alexa Fluor 488-labelled early- or late-melted *nifH* promoter**. Hydrodynamic radii for σ^{54} and $E\sigma^{54}$ were calculated using Alexa Fluor 488-labelled 463C variant of σ^{54}.

Molecular species	Mean(in nm)±SD
σ^{54}	2.7±0.2
$E\sigma^{54}$	5.3±0.3
**Early-melted Promoter**	
**488-EM**	2.9±0.4
$E\sigma^{54}$**+EM**	6.1±0.6
$E\sigma^{54}$**+EM+PspF**	5.7±0.4
$E\sigma^{54}$**+EM+PspF+ADP.AlFx**	6.2±0.9
$E\sigma^{54}$**+EM+PspF+ATP**	6.2±0.6
**Late Melted Promoter**	
**488-LM**	3.3±0.2
$E\sigma^{54}$**+LM**	5.1±0.2
$E\sigma^{54}$**+LM+PspF**	6.2±0.4
$E\sigma^{54}$**+LM+T86A**	5.0±0.1
$E\sigma^{54}$**+LM+PspF+ADP.AlFx**	6.4±0.7
$E\sigma^{54}$**+LM+PspF+ATP+hep**	4.9±0.4
**RPo+UpG**	4.5±0.5
**RPo+UpG+GTP**	4.2±0.1
**RPo+UpG+NTP**	3.9±0.1
**Off-promoter remodeling**	
σ^{54}**+PspF+ATP**	3.1±0.2
$E\sigma^{54}$**+PspF+ATP**	5.6±0.3

Table S4: **List of FRET efficiencies and distances measured for the different molecular complexes using DL and singly-labelled variants (463C or 20C) of σ^{54}.**

Figure	Molecular Species	FRET Population	E mean±SD	R(Å) mean±SD
**2(A)**	σ^{54}	Low	0.488(±0.018)	**61(±0.72)**
		High	0.735(±0.023)	**51(±0.98)**
**2(B)**	$E\sigma^{54}$	High	0.710(±0.032)	**52(±0.75)**
**2(C)**	$E\sigma^{54}$+EM	High	0.752(±0.006)	**50(±0.15)**
**2(D)**	$E\sigma^{54}$+EM+PspF	High	0.745(±0.004)	**50(±0.12)**
**2(E)**	$E\sigma^{54}$+EM+PspF+ATP	High	0.769(±0.005)	**49(±0.12)**
**3A**	$E\sigma^{54}$+LM	Low	0.295(±0.011)	**69(±0.36)**
		High	0.734(±0.017)	**51(±0.41)**
**3B**	$E\sigma^{54}$+LM+PspF	Low	0.271(±0.012)	**71(±2.1)**
		High	0.744(±0.034)	**50(±0.85)**
**3C**	RPi	Low	0.135(±0.015)	**82(±1.00)**
		High	0.736(±0.017)	**51(±0.43)**
**3D**	RPo	Low	0.221(±0.01)	**74(±0.39)**
		High	0.707(±0.015)	**52(±0.19)**
**4(A-F)**	RPo	Low	0.221(±0.008)	**74(±0.39)**
		High	0.707(±0.015)	**52(±0.19)**
**5(A)**	RPo+UpG	Low	0.219(±0.01)	**74(±0.62)**
		High	0.695(±0.013)	**52(±0.11)**
**5(B)**	RPo+UpG+GTP	Low	0.215(±0.005)	**75(±0.68)**
		High	0.704(±0.005)	**52(±0.33)**
**5(C)**	RPo+UpG+NTPs	Low	0.230(±0.016)	**73(±1.1)**
		High	0.708(±0.004)	**52(±0.14)**
**S3(C)**	Denatured σ^{54}	Low	0.080(±0.011)	**90(±0.45)**

**S4(A)**	Eσ^{54}+LM+heparin	High	0.691(±0.010)	**53(±0.22)**
**S4(B)**	Eσ^{54}+LM+PspF(T86A)	Low<br>High	0.292(±0.012)<br>0.740(±0.022)	**71(±0.10)**<br>**49(±0.30)**
**S5A(top)**	Eσ^{54}+LM	Low	0.499(±0.01)	**60(±1.0)**
**S5A (second)**	Eσ^{54}+LM+PspF	Low	0.478(±0.01)	**61(±1.0)**
**S5A (third)**	RPi	Low<br>High	0.512(±0.01)<br>0.722(±0.01)	**60(±1.0)**<br>**51(±1.0)**
**S5A (bottom)**	RPo	High	0.801(±0.01)	**48(±1.0)**
**S5B(top)**	Eσ^{54}+LM	Low	0.637(±0.01)	**60(±1.0)**
**S5B(second)**	Eσ^{54}+LM+PspF	Low	0.622(±0.01)	**61(±1.0)**
**S5B(third)**	RPi	Low	0.560(±0.01)	**61(±1.0)**
**S5B(bottom)**	RPo	Low	0.417(±0.01)	**63(±1.0)**
**S6(A)**	σ^{54}(R336A)	High	0.727(±0.006)	**51(±0.24)**
**S6(B)**	Eσ^{54}(R336A)	High	0.709(±0.013)	**52(±0.13)**
**S6(C)**	Eσ^{54}(R336A)+LM	Low<br>High	0.231(±0.003)<br>0.678(±0.006)	**73(±0.21)**<br>**53(±0.24)**
**S7(A)top**	σ^{54}+PspF	High	0.710((±0.008)	**52(±0.5)**
**S7(A)bottom**	σ^{54}+PspF+ATP	High	0.725(±0.008)	**51(±0.32)**
**S7(B) top**	Eσ^{54}+PspF	High	0.69(±0.013)	**53(±0.53)**
**S7(B) bottom**	Eσ^{54}+PspF+ATP	High	0.714(±0.013)	**52(±0.52)**
